# [Retracted] Resveratrol attenuates type 2 diabetes mellitus by mediating mitochondrial biogenesis and lipid metabolism via Sirtuin type 1

**DOI:** 10.3892/etm.2026.13227

**Published:** 2026-07-01

**Authors:** Ming-Ming Cao, Xi Lu, Guo-Dong Liu, Ying Su, Yan-Bo Li, Jin Zhou

Exp Ther Med 15:576–584, 2018; DOI: 10.3892/etm.2017.5400

Following the publication of the above paper, it was drawn to the Editor’s attention by a concerned reader that western blot data featured in Fig. 1B and 3B on p. 580 and 581 respectively were strikingly similar to data that appeared in another article submitted to the journal *Molecular Medicine Reports* approximately one month after this paper’s receipt at *Experimental and Therapeutic Medicine*, even though the papers were written by different research groups at different institutes. In addition, an independent analysis of the data in the paper undertaken by the Editorial Office revealed the apparent re-use of control western blot data, comparing Figs. 1B and 3B.

Given the issue of data re-use and the promixity of the submission of this paper to *Experimental and Therapeutic Medicine* with that of the other paper to *Molecular Medicine Reports* that was written by different authors, the Editor has decided that this paper should be retracted from the Journal on account of a lack of confidence in the presented data. After contacting the authors, they accepted the decision to retract the paper. The Editor apologizes to the readership for any inconvenience caused.

